# Decreased antiviral immune response within the central nervous system of aged mice is associated with increased lethality of West Nile virus encephalitis

**DOI:** 10.1111/acel.13412

**Published:** 2021-07-30

**Authors:** Kristen E. Funk, Artem D. Arutyunov, Pritesh Desai, James P. White, Allison L. Soung, Sarah F. Rosen, Michael S. Diamond, Robyn S. Klein

**Affiliations:** ^1^ Department of Internal Medicine Division of Infectious Diseases Washington University School of Medicine Saint Louis Missouri USA; ^2^ Center for Neuroimmunology and Neuroinfectious Diseases Washington University School of Medicine Saint Louis Missouri USA; ^3^ Department of Molecular Microbiology Washington University School of Medicine Saint Louis Missouri USA; ^4^ Department of Pathology and Immunology Washington University School of Medicine Saint Louis Missouri USA; ^5^ Department of Neurosciences Washington University School of Medicine Saint Louis Missouri USA; ^6^ Present address: Department of Biological Sciences University of North Carolina at Charlotte Charlotte North Carolina USA

**Keywords:** aging, antiviral T cells, CNS, microglia, viral encephalitis, West Nile virus

## Abstract

West Nile virus (WNV) is an emerging pathogen that causes disease syndromes ranging from a mild flu‐like illness to encephalitis. While the incidence of WNV infection is fairly uniform across age groups, the risk of lethal encephalitis increases with advanced age. Prior studies have demonstrated age‐related, functional immune deficits that limit systemic antiviral immunity and increase mortality; however, the effect of age on antiviral immune responses specifically within the central nervous system (CNS) is unknown. Here, we show that aged mice exhibit increased peripheral organ and CNS tissue viral burden, the latter of which is associated with alterations in activation of both myeloid and lymphoid cells compared with similarly infected younger animals. Aged mice exhibit lower MHCII expression by microglia, and higher levels of PD1 and lower levels of IFNγ expression by WNV‐specific CD8^+^ T cells in the CNS and CD8^+^CD45^+^ cells. These data indicate that the aged CNS exhibits limited local reactivation of T cells during viral encephalitis, which may lead to reduced virologic control at this site.

## Graphical abstract

West Nile virus (WNV) is an emerging pathogen with a higher risk of lethal encephalitis in individuals with advanced age. Here, we show that aged mice exhibit increased peripheral organ and CNS tissue viral burden, the latter of which is associated with alterations in activation of both myeloid and lymphoid cells compared with similarly infected younger animals.

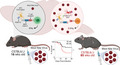

## 1 INTRODUCTION

West Nile virus (WNV), a mosquito‐transmitted RNA virus belonging to the *Flaviviridae* family, is responsible for recurring outbreaks of meningitis and encephalitis each summer in the United States and parts of Eastern and Southern Europe (Chancey et al., 2015). The majority of WNV infections are asymptomatic (~80%); however, some infected patients develop a symptomatic flu‐like illness (~20%), up to half of which may progress to neuroinvasive forms of central nervous system (CNS) infection including meningitis, encephalitis, and acute flaccid paralysis. Older age is the most well‐defined risk factor for developing neuroinvasive illness, with an approximately 20‐fold increased risk (Final Cumulative Maps and Data | West Nile Virus | CDC, 2019). Despite the recognition of advanced age as a risk factor, the neuroimmune correlates underlying susceptibility of the elderly to lethal WNV infection are not well understood.

Animal studies indicate that clearance of WNV in peripheral organs requires coordinated response of multiple aspects of the immune system including antiviral cytokines (Samuel & Diamond, 2005), complement activation (Mehlhop et al., 2005), neutralizing antibody induction (Diamond et al., 2003), CD4^+^ T cell helper, effector, and regulatory functions (Graham et al., 2014; Lanteri et al., 2009), and CD8^+^ T cell cytotoxic functions (Brien et al., 2007). Aging is associated with distinct changes in many of these immune cell populations and a progressive decline in immune function, leading to increased susceptibility to infections (Carr et al., 2016). Previous studies of WNV infection in mice aged 18–22 months observed increased viral burden in the CNS compared with younger 4–6‐month‐old mice (Brien et al., 2009; Richner et al., 2015). Brien et al. demonstrated that loss of virological control correlated with age‐related defects in peripheral CD4^+^ and CD8^+^ T cell responses, including deficient cytokine and lytic granule production, and 10‐fold fewer total effector CD8^+^ T cells in the brains of aged versus adult mice during acute infection (Brien et al., 2009). Richner *et al*. detected cell‐intrinsic defects in naïve CD4^+^ T cells as well as reduced cytokine and chemokine levels within draining lymph nodes (DLN), both of which contributed to reduced immune cell accumulation, delayed germinal center development, and decreased immune responses in aged mice compared with younger controls (Richner et al., 2015). These age‐dependent defects occurred within the first few days of infection, resulting in failure to control WNV infection in peripheral tissues and increased neuroinvasion and lethality in aged mice (Richner et al., 2015). However, neither of these studies investigated the effects of aging on CNS myeloid cells or T cell subsets.

CNS myeloid cells, which include brain‐resident microglia and infiltrating monocytes, are innate immune cells that become activated in response to danger signals. During WNV infection, virally infected neurons secrete chemokines including CCL2, CCL5, and CXCL10, that facilitate entry of monocytes and lymphocytes into the CNS (Durrant et al., 2014; Klein et al., 2005). The CNS recruitment of monocytes, which differentiate into tissue macrophages at specific sites of infection, is critical for controlling WNV replication in the CNS. Macrophages that infiltrate the infected CNS amplify the chemokine signal to further recruit and stimulate antiviral T cells (Getts et al., 2012), and restrict the accumulation of other inflammatory leukocytes that may contribute to immunopathology and prevent healing (London et al., 2011; Shechter et al., 2009). Microglia also coordinate innate and adaptive immune functions in response to infection (Funk & Klein, 2019). Elimination of microglia with a CSF1R antagonist increases the susceptibility of mice to lethal WNV infection by limiting local restimulation of antiviral CD8^+^ T cells once they enter the CNS parenchyma (Funk & Klein, 2019).

Here, we assessed the impact of advanced age on the response of myeloid cells to CNS infection using a mouse model of WNV. Our results show that aged mice are more susceptible to lethal WNV infection, which correlates with impaired immune response and increased viral titers in the CNS. Our results show that both prior to and during acute infection, a greater percentage of splenic T cells isolated from 85‐week‐old versus 16 week mice express inhibitory immune checkpoint marker PD1. This was associated with decreased WNV‐specific CD8^+^ T cells isolated from the spleens of 85 week mice during acute infection and increased dissemination of WNV to the CNS. Within the CNS, cortical microglia in 85 week mice did not expand in response to infection, unlike in similarly infected 16 week animals. Greater numbers of microglia isolated from cortices of infected 16 week animals expressed the phagocytic marker CD68, as well as antigen presentation molecule MHCII compared with microglia from infected animals. We also found fewer CD8^+^ T cells and fewer WNV‐specific CD8^+^ T cells in the cortices of WNV‐infected 85 week aged animals compared with infected 16 week animals. Of the WNV‐specific CD8^+^ T cells isolated from 85 week cortices, fewer cells expressed IFNγ and at lower levels than those isolated from 16 week cortices, suggesting antiviral responses are decreased in the CNS of aged animals. We also identified a population of age‐related myeloid cells in uninfected 85 week mice that are CD11b^+^CD45^lo^, similar to resting microglia, but do not express microglial marker P2RY12. These cells are absent from uninfected 16 week cortices, but are present in 16 week cortices after viral clearance, and similar to myeloid cells isolated from 85 week cortices, express CD68 and MHCII. Together, these data suggest that aging impacts the antiviral response of microglia and that viral infection may impact aging processes of CNS resident microglia.

## 2 RESULTS

### 2.1 Aged mice are more susceptible to lethal WNV infection despite virologic control in peripheral organs

**FIGURE 1:**
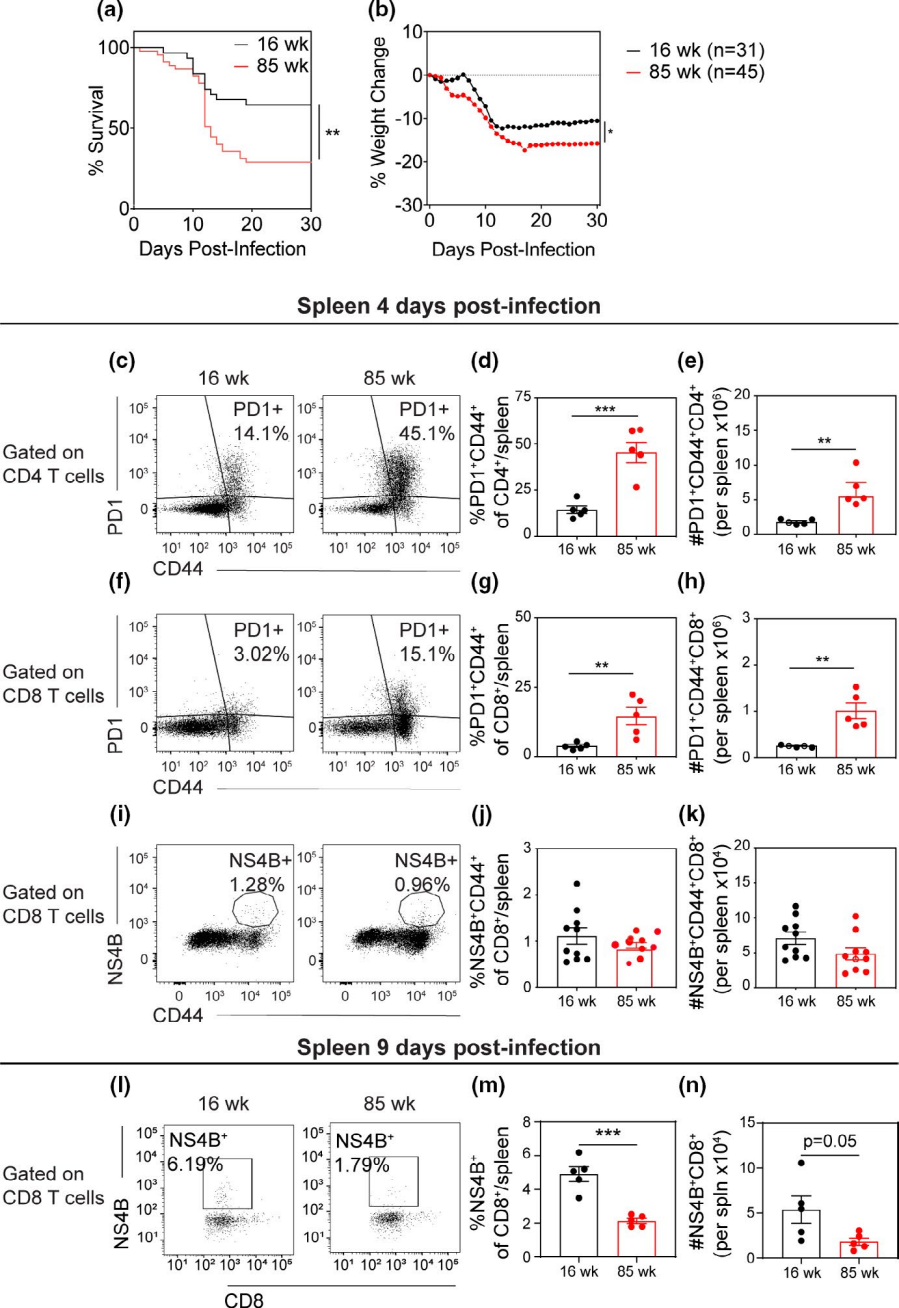
Aged mice show reduced CD8^+^ T cell priming in spleen during acute infection and are more susceptible to lethal viral challenge compared to adult mice. (a, b) 16 week (black) and 85 week (red) mice underwent footpad (f.p.) inoculation of 10^2^ PFU WNV‐NY, then monitored for (a) mortality and (b) weight loss. (a) Survival curves show a significant increase in mortality in aged mice compared with 16 week mice, as calculated by Log‐rank (Mantel–Cox) test. (b) Weight was measured daily during acute illness as an indicator of illness. After death, the last measured weight was carried through to the end of the experiment. Aged mice lost a significantly greater percentage of baseline weight compared with 16 week mice, as calculated by two‐way ANOVA with matched values comparing group means. (c‐k) At 4 dpi or (l‐n) 9 dpi, splenocytes were isolated and phenotyped by flow cytometric analysis. (c) At 4 dpi, representative flow cytometry plots of CD4^+^‐gated cells analyzed for PD1 and CD44 expression in 16 week (*left*) and 85 week (*right*) mice. (d, e) Quantification of (d) percent and (e) number PD1^+^CD44^+^ of CD4^+^ T cells per spleen. (f) Representative flow cytometry plots of CD8^+^‐gated cells analyzed by PD1 vs CD44. (g, h) Quantification of (g) percent and (h) number of PD1^+^CD44^+^ of CD8^+^ T cells per spleen. For panels A‐F, data are representative of 1 experiment with 5 mice per group. (i) Representative flow cytometry plots of CD8^+^‐gated cells analyzed by WNV‐specific tetramer NS4B vs CD44. (j, k) Quantification of (j) percent and (k) number of NS4B^+^CD44^+^ of CD8^+^ T cells per spleen. For panels I‐K, data are a compilation of 2 experiments with 10 mice per group. Statistical significance was calculated by unpaired *t* test. (l) At 9 dpi, representative flow cytometry plots of CD8^+^‐gated cells analyzed by WNV‐specific tetramer NS4B vs CD44. (m, n) Quantification of (m) percent and (n) number of NS4B^+^ of CD8+ T cells per spleen. For Panels L‐N, data are representative of 1 experiment with 5 mice per group. For panels C‐K, statistical significance was calculated by *t* test

Because increased age and male sex is associated with higher rates of WNV neuroinvasive disease and lethality in humans, we evaluated disease severity following infection with WNV‐NY in young (16 week) and aged (85 week) C57BL/6J male mice. Aged mice exhibited increased lethality and greater weight loss than 16 week mice (Figure 1a,b). To determine whether increased lethality is associated with loss of virological control in peripheral organs, we assessed viral titers in the serum (Figure S1A), spleen (Figure S1B), and kidney (Figure S1). We detected increased WNV infection in the kidney at day 6 post‐infection (dpi) and viremia at 8 dpi in 85 week versus 16 week mice. However, virus declined or was cleared from each of these compartments by 9 dpi, the time point just prior to when mice begin succumbing to infection. This suggests that the increased lethality of WNV infection in 85 week mice may be more complex than just lack of virological control in peripheral organs.

### 2.2 Aged mice have limited antiviral T cell responses in peripheral immune organs

Previous studies have demonstrated increased expression of immune checkpoint inhibitor molecules on T cells isolated from aged mice, which is associated with decreased immune responses to pathogenic insults (Lee et al., 2016). Our data support this hypothesis showing that CD4^+^ and CD8^+^ T cells isolated from uninfected spleens (Figure S2) and inguinal lymph nodes (Figure S3) in 85 week versus 16 week mice have greater number and percentage of PD1^+^ T cells. This difference is maintained during acute WNV infection, with increased numbers and percentages of PD1^+^CD4^+^ T cells (Figure 1c–e) and PD1^+^CD8^+^ T cells (Figure 1f–h) isolated from 85 week versus 16 week spleen at 4 dpi. A similar trend in PD1^+^ T cells was seen in the draining inguinal lymph node but did not reach statistical significance (Figure S4). Given that PD1 expression may limit the priming and clonal expansion of WNV‐specific CD8^+^ T cells, we examined the generation of antiviral T cells in both cohorts. At 4 dpi, we found no substantive difference in the number or percentage of CD8^+^ T cells specific to the immunodominant WNV NS4B peptide; however, at 9 dpi we found a significant deficit in both the number and the percentages of NS4B^+^CD8^+^ T cells isolated from 85 week versus 16 week spleens (Figure 1l–n).

Previous studies have indicated that CD8^+^ T cells contribute to acute gastrointestinal (GI) dysmotility following WNV infection (White et al., 2018). Because advanced age appears to limit CD8^+^ T cell function, we tested whether aging affects the GI dysmotility phenotype. Passage of carmine red dye in feces of mock‐ and WNV‐infected mice was measured at 10 dpi. As seen in previous studies (White et al., 2018), 16 week mice showed delayed transit at 10 dpi (Figure S1D). Although GI transit in infected 85 week mice was significantly delayed compared to mock‐infected age‐matched controls, the delay was less than in the infected 16 week mice (Figure S1D). Despite the differences in GI motility, we found no significant difference between 16 week and 85 week mice in viral titers in either the distal small intestine (Figure S1E) or the colon (Figure S1F). The 85 week aged mice may paradoxically show less GI dysmotility in this model than younger mice because of their impaired T cell responses.

### 2.3 Aged mice have increased viral titers and decreased antiviral immune response in the CNS during acute infection

**FIGURE 2:**
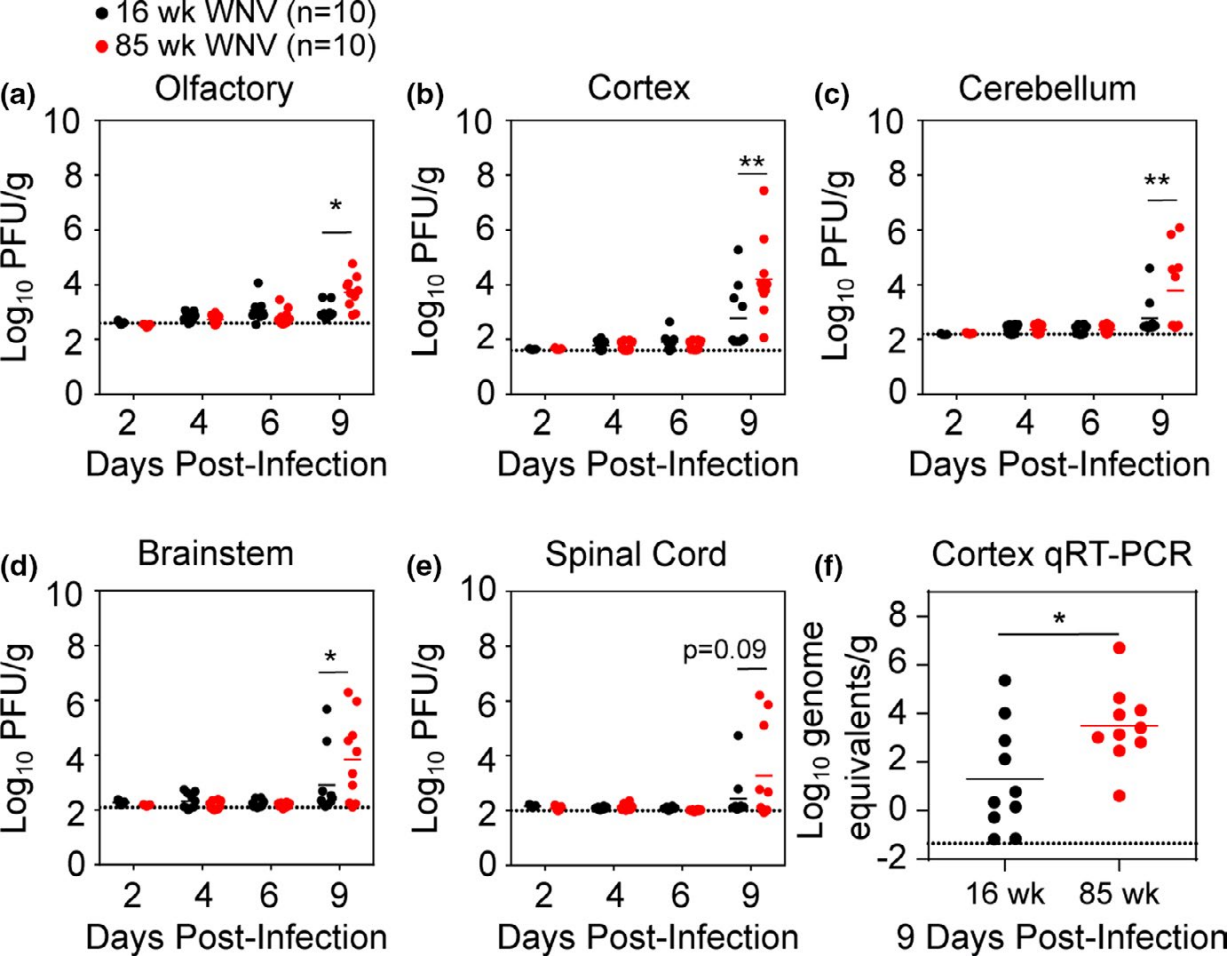
Aged mice have increased CNS viral burden. (a–e) CNS viral loads were measured by plaque assay at 2, 4, 6, and 9 dpi following inoculation with 10^2^ PFU in 16 week (black) and 85 week (red) mice. Statistical significance was calculated using two‐way ANOVA with Sidak's multiple comparisons test. (f) Cerebral cortex viral genome equivalents were measured by qRT‐PCR. Statistical significance was calculated unpaired *t* test. Data are presented as scatter plots with lines indicating mean. Dotted lines indicate assay limit of detection. These data are the compilation of 2 independent experiments with 10 mice per group. *p < 0.05; **p < 0.01; ****p < 0.0001

Because lethal WNV infection in mice is associated with dissemination to the CNS, we quantified viral titers in the olfactory bulb, cortex, cerebellum, brainstem, and spinal cord using standard plaque assay. At 9 dpi, a time point just prior to when mice begin to succumb to infection, 85 week mice had greater viral titers than 16 week mice in all CNS tissues except for the spinal cord (Figure 2a‐e). To assess viral titers in the cortex that might have fallen below the limit of detection of plaque assay, we quantified viral RNA levels by qRT‐PCR at 9 dpi. This analysis also showed increased viral titers in the 85 week cortex compared to 16 week cortex (Figure 2f).

**FIGURE 3:**
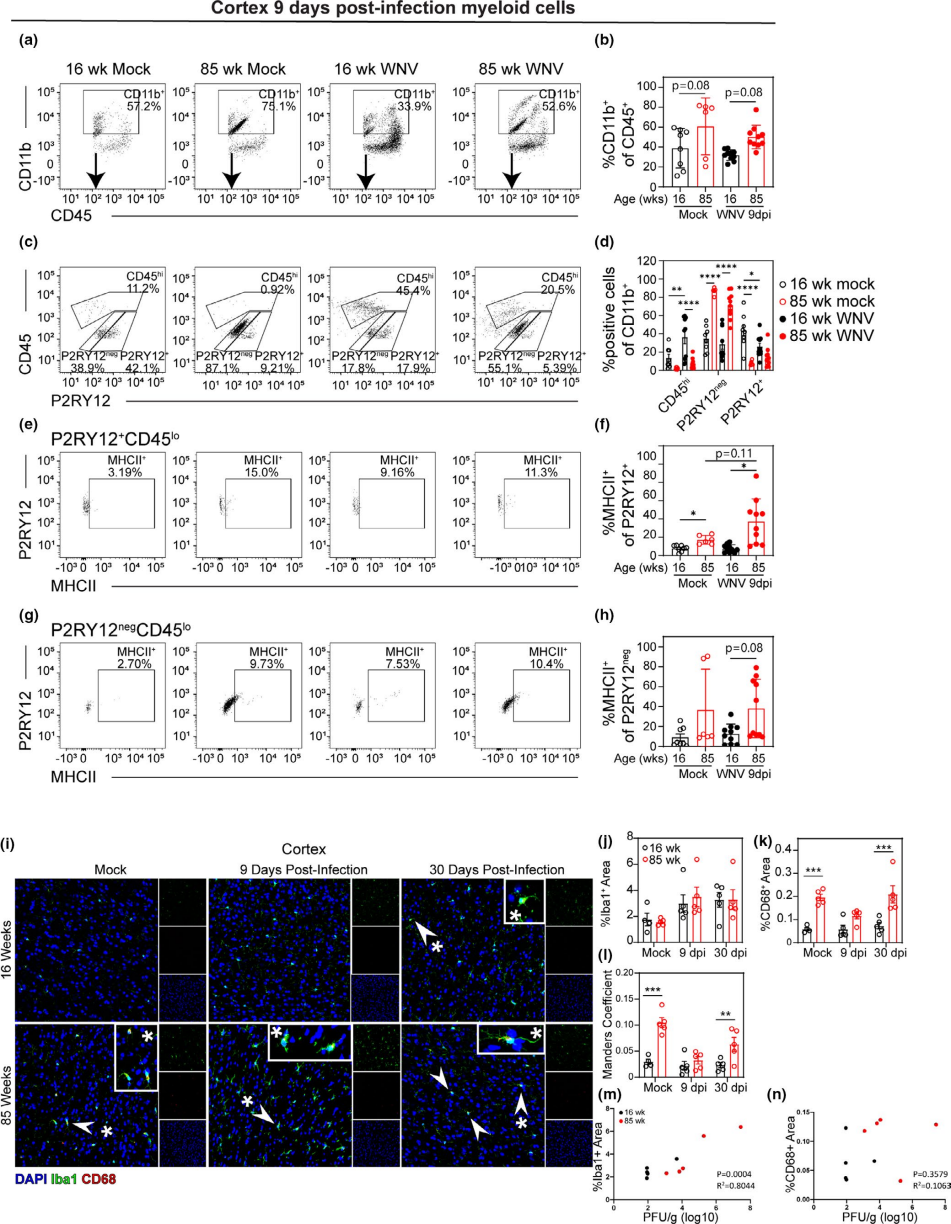
Uninfected aged mice exhibit myeloid cells that lack typical microglial markers. Adult (16 week) and aged (85 week) mice were infected with 100 PFU WNV‐NY via footpad inoculation. At 9 dpi, a time point just prior to when mice begin succumbing to viral infection, leukocytes were isolated from the cortex and analyzed by flow cytometry. (a) Representative flow cytometry plots of CD45^+^‐gated cells analyzed by CD11b vs CD45 expression. (b) Quantification of percent of CD11b^+^CD45^+^ cells in 16 week mock, 85 week mock, 16 week infected, and 85 week infected cortex. (c) Representative flow cytometry plots of CD11b^+^‐gated cells analyzed by CD45 vs P2RY12 expression. (d) Quantification of percent CD45^hi^, P2RY12^+^CD45^lo^, and P2RY12^neg^CD45^lo^ cells in each group. (e) Representative flow cytometry plots of P2RY12^+^CD45^lo^‐gated cells analyzed by P2RY12 vs MHCII expression. (f) Quantification of percent MHCII^+^ of P2RY12^+^CD45^lo^‐gated cells in each group. (g) Representative flow cytometry plots of P2RY12^neg^CD45^lo^‐gated cells analyzed by P2RY12 vs MHCII expression. (h) Quantification of percent MHCII^+^ of P2RY12^neg^CD45^lo^‐gated cells in each group. These data are representative of two experiments with 6–10 mice per group. Statistical significance was calculated using one‐way ANOVA with Welch's correction and Dunnett's multiple comparisons test. (i) Representative images of 16 week (*top row*) and 85 week (*bottom row*) cortical tissue sections collected from mock‐ (*left*) or WNV‐infected mice at 9 dpi (*middle*), or 30 dpi (*right*). Sections were immunostained with antibodies to Iba1 (green) and CD68 (red), and counterstained with DAPI (blue). Arrowheads point to colocalization of Iba1 and CD68. Asterisks denote regions magnified in inset panels. (j–l) Quantification of (j) percent area covered by Iba1 staining, (k) percent area covered by CD68 staining, and (l) Manders coefficient of colocalization between CD68 and Iba1 and calculated by ImageJ. Statistical significance was calculated by two‐way ANOVA with Sidak's multiple comparison test. *p < 0.05; **p < 0.01; ***p < 0.001; ****p < 0.0001

To determine whether age affects the antiviral response of myeloid cells within the CNS, including resident microglia and infiltrating macrophages, we isolated immune cells from the cortices of 85 week and 16 week mice at 9 dpi and phenotyped them by flow cytometry (Figure S5). In uninfected mice, the cerebral cortices of 85 week had greater percentage of CD11b^+^CD45^+^ cells than 16 week cortices, although this difference was not statistically significant (Figure 3a,b). CD11b^+^CD45^+^ cells were further characterized for levels of CD45. Only mice infected with WNV accumulated appreciable numbers of CD11b^+^CD45^hi^ cells, which include peripheral myeloid cells that infiltrate the CNS (Veremeyko et al., 2012); however, we identified even fewer of these cells in 85 week versus 16 week infected mice (Figure 3c,d).

To assess microglial numbers, cells were immunostained for P2RY12, which is a phenotypic marker expressed by resident microglia but not infiltrating macrophages (Butovsky et al., 2014). Both 16 week and 85 week mice exhibited CNS populations of CD11b^+^CD45^lo^ cells that were either P2RY12^neg^ or P2RY12^+^, but uninfected 85 week mice had significantly greater numbers of P2RY12^neg^CD11b^+^CD45^lo^ cells than uninfected 16 week mice in their cerebral cortices (Figure 3c,d). Similarly, the number of P2RY12^neg^CD11b^+^CD45^lo^ cells isolated from the cortices of 85 week mice at 9 dpi was significantly greater than the numbers isolated from the cortices of 16 week mice at 9 dpi (Figure 3c,d). Given that downregulation of P2RY12 occurs during CNS injury in older mice (Klein et al., 2018), it is possible that these cells are microglia. Unexpectedly, compared with those isolated from the cortices of 16 week mock‐infected mice, a lower percentage of P2RY12^+^ cells were isolated from the cortices of both 85 week mock‐infected and 16 week WNV‐infected mice at 9 dpi (Figure 3c,d), further supporting the notion that P2RY12^neg^ cells may also be microglia that have downregulated P2RY12. While *in vitro* studies suggest P2RY12 may be associated with anti‐inflammatory microglia (Moore et al., 2015), its roles during CNS aging and viral infection are unknown. Together, these data indicate that although aged mice have greater numbers of myeloid cells at baseline, these numbers decrease following viral infection.

To further investigate the activation state of myeloid cells in the brain, we examined MHCII expression on P2RY12^+^CD11b^+^CD45^lo^ and P2RY12^neg^CD11b^+^CD45^lo^ cells. Myeloid cells would be expected to upregulate MHCII in response to viral infection (Tsai et al., 2016). Our results show that a greater percentage of P2RY12^+^CD11b^+^CD45^lo^ cells isolated from 85 week cortices are MHCII+compared with cells isolated from 16 week cortices in both mock‐infected and WNV‐infected groups (Figure 3e,f). We also saw a nonsignificant increase in the percentage of MHCII^+^P2RY12^neg^CD11b^+^CD45^lo^ cells isolated from the cortices of WNV‐infected 85 week mice versus infected 16 week mice (Figure 3g,h). The lack of upregulation in 16 week mice is likely due to low CNS viral loads at this time point.

Myeloid cell activation was also assessed in the cerebral cortex (Figure 3i‐l), hippocampus (Figure S6A‐D), and cerebellum (Figure S6E‐H) via immunohistochemical staining for Iba1 and CD68 during acute infection (9 dpi) and following viral clearance (30 dpi). Iba1 is a marker for microglia and macrophages (Ito et al., 1998), and CD68 is a marker of phagocytic activity in microglia and macrophages (Zotova et al., 2013). 85 week mice had increased staining of CD68 compared to 16 week mice in both mock‐infected and at 30 dpi in the cortices (Figure 3i,k) and cerebella (Figure S6E and G), although there was no significant difference in Iba1 staining (Figure 3F and Figure S6J). All three brain regions investigated (cortex, hippocampus, and cerebellum) showed increased colocalization between Iba1 and CD68 in 85 versus 16 week brains at 30 dpi, as measured by Manders coefficient (Figure 3l, Figure S6D and Figure S6H). 85 week mice also had increased colocalization between CD68 and Iba1 in the cerebral cortices of mock‐infected animals (Figure 3l). Together, these data suggest that microglia in 85 week mice exhibit increased activation at baseline and after recovery from WNV infection.

Because neuronal death may contribute to lethality, we assessed cell death due to viral infection within the CNS of WNV‐infected 16 week versus 85 week mice via detection of TUNEL staining at 9 dpi and compared it to mock‐infected animals. Quantitation of TUNEL‐positive puncta in the hippocampi (Figure S7A and B), cerebral cortices (Figure S7C and D), and cerebella (Figure S7E and F) showed statistically significant increased TUNEL staining in 85 week versus 16 week cerebella and hippocampi. Co‐occurrence analysis showed that both neurons and microglia are affected by cell death in all three brain regions studied (Figure S7C,F,I), with the two cell types often found in close proximity. In the hippocampus, WNV infection significantly increased the number TUNEL‐positive staining independently of age, which was different from the cerebellum, where advanced age alone leads to an increase in cell death.

To determine whether increased viral titers in the CNS of 85 week mice correlated with antiviral T cell response, we isolated leukocytes from the cerebral cortices of 16 week and 85 week mice at 9 dpi. We identified a significant increase in the number of lymphocytes (Figure S8A and B), CD8^+^ T cells (Figure S8C and D), and NS4B^+^CD8^+^ T cells (Figure S8E and F) of 16 week mice at 9 dpi compared to mock‐infected age‐matched controls. We also saw a nonsignificant increase in the number of IFNγ^+^CD8^+^ T cells (Figure S8G and H) isolated from the cortices of 16 week mice versus mock‐infected age‐matched controls. There were also significantly fewer NS4B^+^CD8^+^ T cells isolated from the cortices of 85 week compared with 16 week infected mice (Figure S8E and F). Overall, the increased number of antiviral T cells was expected, based on previous reports (Brien et al., 2009). However, it is notable that the relative increase in the number of IFNγ^+^CD8^+^ T cells isolated from young infected mice is less robust than the number of CD8^+^ T cells or NS4B^+^CD8^+^ T cells, which reflect the lack of MHCII upregulation seen in these mice.

### 2.4 Diminished antiviral immunity in the CNS of aged mice persists even after virus has cleared

**FIGURE 4:**
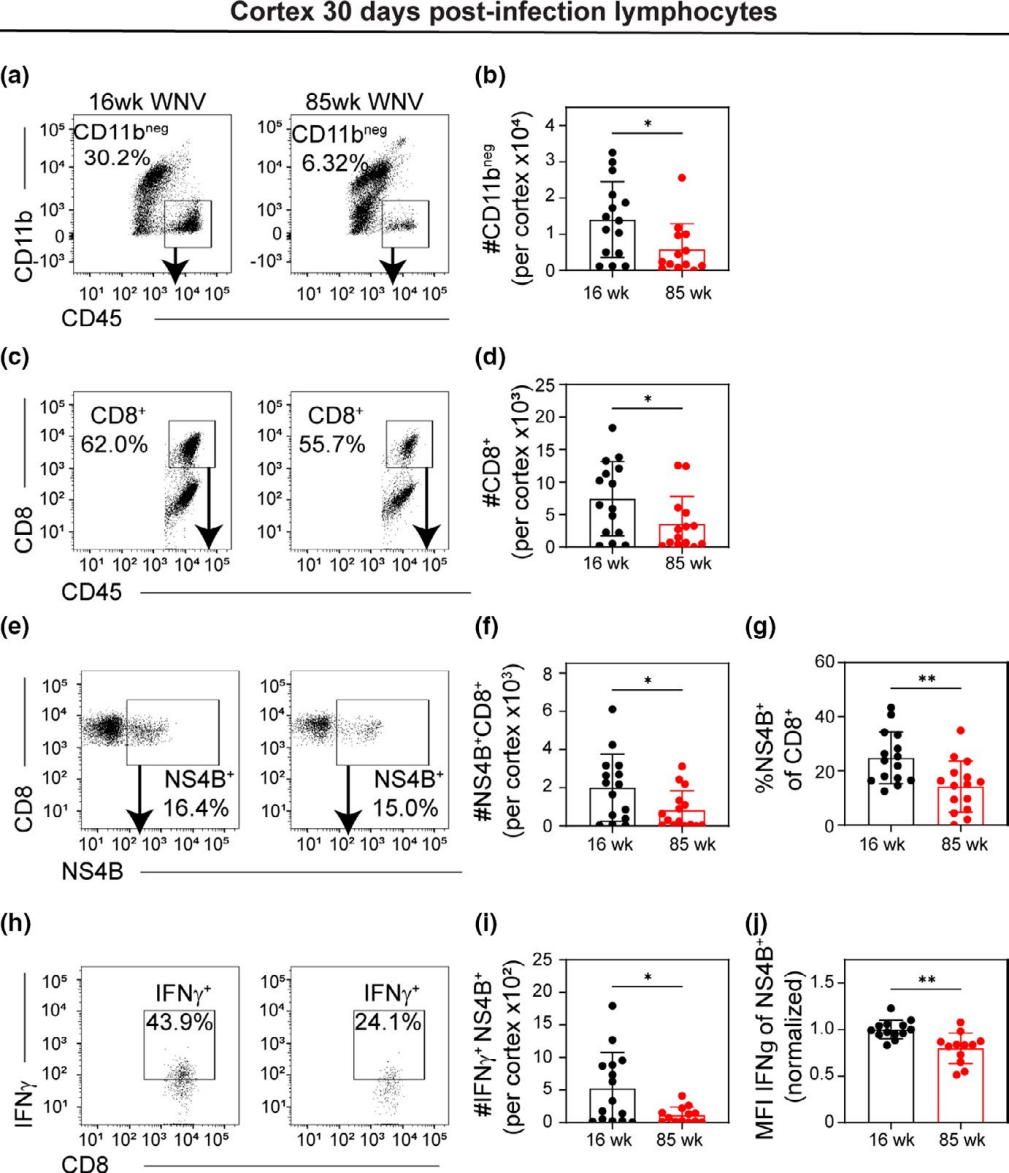
Surviving aged mice show exhausted T cell phenotype in CNS. 16 week and 85 week mice were infected with 100 PFU WNV‐NY via footpad inoculation. At 30 dpi, mice that survived the infection were euthanized, and leukocytes were isolated from the cortex and analyzed by flow cytometry. (a) Representative flow cytometry plots of CD45^+^‐gated cells analyzed by CD11b vs CD45 of 16 week (*left*) and 85 week (*right*) at 30 dpi. (b) Quantification of number of CD11b^neg^CD45^+^ cells in each group. (c) Representative flow cytometry plots of CD11b^neg^‐gated cells analyzed by CD8 vs CD45 staining. (d) Quantification of number of CD8^+^CD45^+^ cells in each group. (e) Representative flow cytometry plots of CD8^+^‐gated cells analyzed by CD8 vs NS4B (WNV‐specific tetramer) staining. (f, g) Quantification of (f) number and (g) percent of NS4B^+^ of CD8^+^ cells in each group. (h) Representative flow cytometry plots of NS4B^+^‐gated cells analyzed by IFNγ vs NS4B staining. (i, j) Quantification of (i) number and (j) normalized MFI of IFNγ^+^ of NS4B^+^CD8^+^ cells. MFI was normalized within each experiment to the average IFNγ MFI of NS4B^+^CD8^+^ cells isolated from 16 week mice at 30 dpi. Each mouse is represented by a symbol and lines indicate mean ± SD. These data are the representative of three independent experiments with a total of 9–15 mice per group. Statistical significance was calculated using one‐way ANOVA with Welch's correction and Dunnett's multiple comparisons test. *p < 0.05; **p < 0.01

To determine whether diminished antiviral immunity in the CNS persists in 85 week mice that survive the acute infection, we analyzed cellular immunity at 30 dpi, a time point at which virus has cleared. Lymphocytes were isolated from the cerebral cortices of surviving 16 week and 85 week mice at 30 dpi and analyzed by flow cytometry. Results show that 85 week mice have fewer CD45^hi^CD11b^neg^ (Figure 4a,b) and CD8^+^CD45^+^ cells (Figure 4c,d) within the cerebral cortices than those of 16 week mice. Moreover, a decreased number and percentage of WNV‐specific CD8^+^CD45^+^ cells were isolated from 85 week mice even though these animals on average experienced greater infection (Figure 4e–g). We also found decreased number of NS4B^+^ cells expressing IFNγ (Figure 4h,i), and cells isolated from 85 week mice have lower expression levels (based on normalized median fluorescent intensity (MFI) of staining) upon *ex vivo* restimulation compared with cells isolated from 16 week mice (Figure 4j). Together, these data suggest that fewer WNV‐specific T cells reside in the aged CNS after the virus has cleared, and of the cells that do, they are less poised to respond with antiviral cytokines.

**FIGURE 5:**
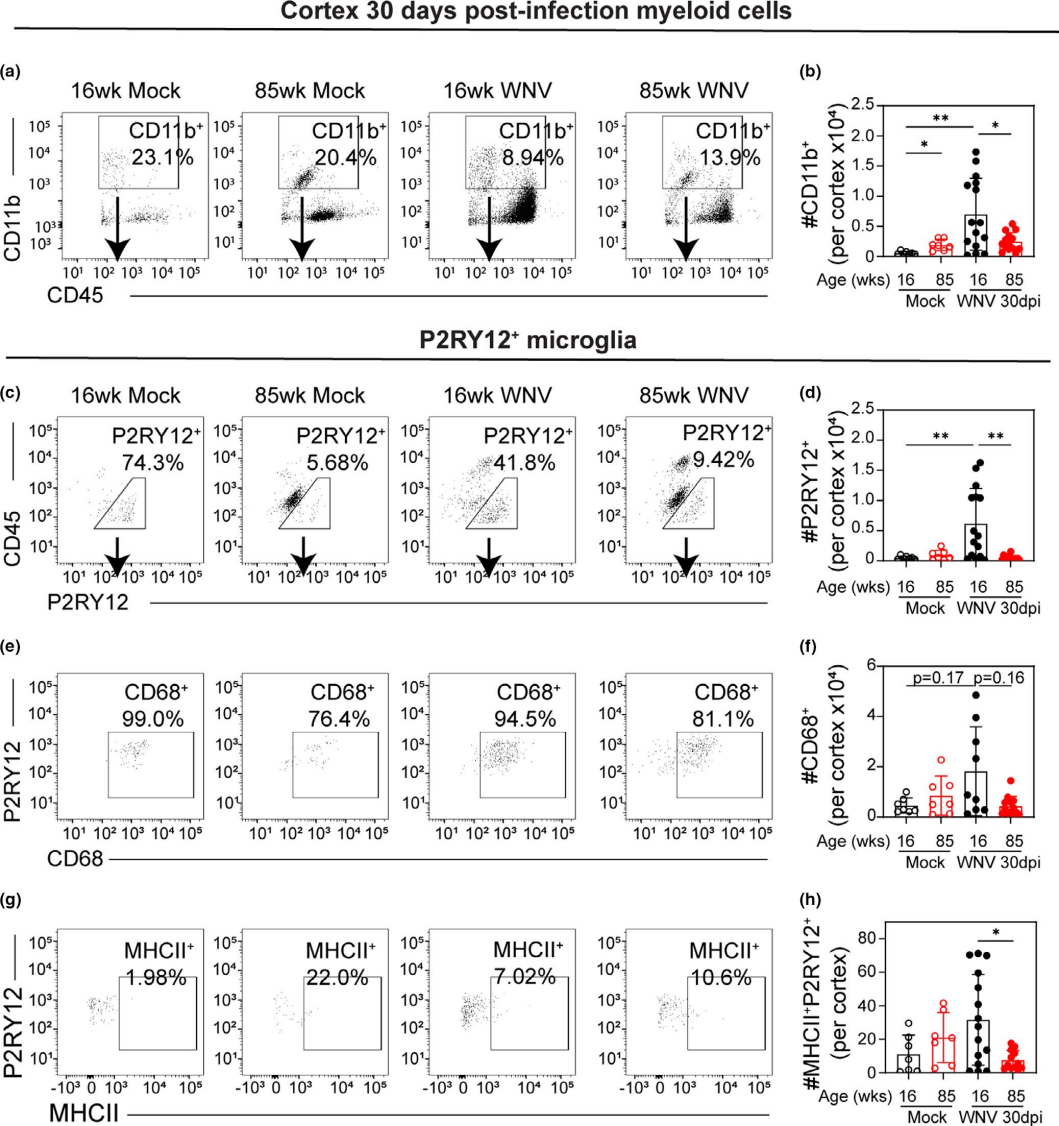
Surviving aged mice show less microglial activation than young adult mice. 16 week and 85 week mice were infected with 100 PFU WNV‐NY via footpad inoculation. At 30 dpi, mice that survived the infection were euthanized, and leukocytes were isolated from the cortex and analyzed by flow cytometry. (a) Representative flow cytometry plots of CD45^+^‐gated cells analyzed by CD11b vs CD45 expression. (b) Quantification of number of CD11b^+^CD45^+^ cells in each group. (c) Representative flow cytometry plots of CD11b^+^‐gated cells analyzed by CD45 vs P2RY12 expression with gating for P2RY12^+^ cells. (d) Quantification of number of P2RY12^+^CD11b^+^ cells in each group. (e) Representative flow cytometry plots of P2RY12^+^‐gated cells analyzed by P2RY12 vs CD68 expression. (f) Quantification of number of CD68^+^P2RY12^+^ cells in each group. (g) Representative flow cytometry plots of P2RY12^+^‐gated cells analyzed by P2RY12 vs MHCII expression. (h) Quantification of number of MHCII^+^P2RY12^+^ cells in each group. These data are the representative of two independent experiments with 7–15 mice per group. Statistical significance was calculated using one‐way ANOVA with Welch's correction and Dunnett's multiple comparisons test. Exact *p* values noted; *p < 0.05; **p < 0.01

**FIGURE 6:**
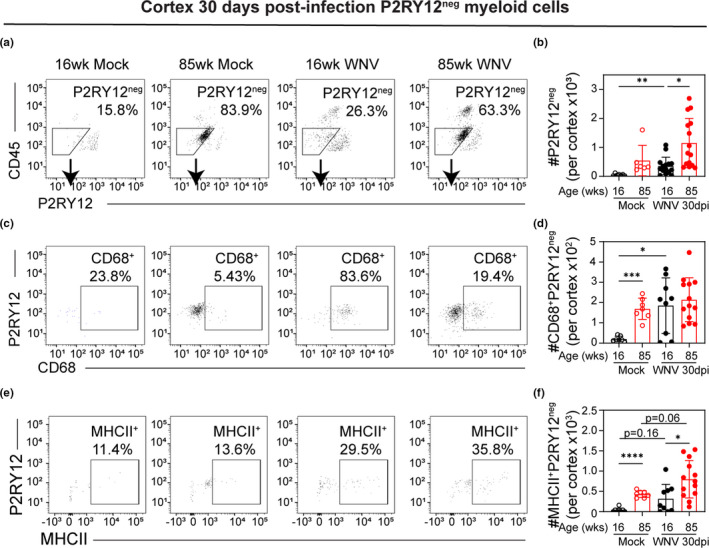
Surviving young mice show increased number of CD45^lo^P2RY12^neg^ myeloid cells. 16 week and 85 week mice were infected with 100 PFU WNV‐NY via footpad inoculation. At 30 dpi, mice that survived the infection were euthanized, and leukocytes were isolated from the cortices and analyzed by flow cytometry. (a) Representative flow cytometry plots of CD11b^+^‐gated cells analyzed by CD45 vs P2RY12 expression with gating for P2RY12^neg^ cells. (b) Quantification of number of P2RY12^neg^CD11b^+^ cells in each group. (c) Representative flow cytometry plots of P2RY12^neg^‐gated cells analyzed by P2RY12 vs CD68 expression. (d) Quantification of number of CD68^+^P2RY12^neg^ cells in each group. (e) Representative flow cytometry plots of P2RY12^neg^‐gated cells analyzed by P2RY12 vs MHCII expression. (f) Quantification of number of MHCII^+^P2RY12^neg^ cells in each group. These data are the representative of two experiment with 7–15 mice per group. Statistical significance was calculated using one‐way ANOVA with Welch's correction and Dunnett's multiple comparisons test. Exact *p* values noted; *p < 0.05; **p < 0.01; ***p < 0.001; ****p < 0.0001

To understand the impact of age on myeloid cells in the CNS after clearance of WNV, we analyzed CD11b^+^CD45^lo^ cells isolated from the cortex at 30 dpi. Notably, the number of CD11b^+^ cells in the cerebral cortices was increased in 16 week but not 85 week mice at 30 dpi relative to age‐matched mock‐infected controls, and fewer CD11b^+^ cells were isolated from 85 week mice versus 16 week mice at 30 dpi (Figure 5a,b). CD11b^+^CD45^lo^ cells were further analyzed by expression of microglial marker P2RY12. Most CD11b^+^CD45^lo^ cells isolated from mock‐infected 16 week mice were P2RY12^+^, whereas most CD11b^+^CD45^lo^ cells isolated from 85 week mice were P2RY12^neg^ (Figures 5c, and 6a). Mirroring the quantification of bulk CD11b^+^ cells, the number of P2RY12^+^CD11b^+^CD45^lo^ cells increased in 16 week but not 85 week mice at 30 dpi relative to mock, and significantly fewer P2RY12^+^CD11b^+^CD45^lo^ cells were isolated from 85 week mice versus 16 week mice at 30 dpi (Figure 5d). Of the P2RY12^+^ cells, most cells were positive for myeloid marker CD68, and although not significant, there were more CD68^+^P2RY12^+^ isolated from 16 week vs 85 week cortex (Figure 5e,f). Also, significantly more P2RY12+MHCII+cells were isolated from the cerebral cortices 16 week versus 85 week mice at 30 dpi (Figure 5g,h). Together, these data indicate that the microglia population in the cerebral cortices of 16 week mice, but not 85 week mice, expands in response to viral infection and that these cells persist after virus has cleared.

Analyzing P2RY12^neg^CD11b^+^CD45^lo^ cells, we found that 85 week mice have greater number of these cells than 16 week WNV‐infected mice at 30 dpi. However, the number of P2RY12^neg^CD11b^+^CD45^lo^ cells increased in 16 week mice at 30 dpi compared to mock (Figure 6a,b). To further analyze these cells, we assessed their expression of CD68, a protein highly expressed by cells of the monocyte lineage and upregulated by phagocytic cells (Chistiakov et al., 2017). Quantitatively, the number of CD68^+^P2RY12^neg^CD11b^+^ cells isolated from the cerebral cortices of mock‐infected 16 week mice is less than either 85 week mock‐infected mice or 16 week mice at 30 dpi (Figure 6c,d). Assessing MHCII expression, a greater number of MHCII^+^P2RY12^neg^ cells were isolated from the cortices of 85 week compared with 16 week mice at 30 dpi as well as from mock‐infected samples (Figure 6e,f). Although not quite significant, more MHCII^+^P2RY12^neg^ were isolated from the cortices of mice at 30 dpi compared to mock‐infected age‐matched samples of both 16 week and 85 week animals (Figure 6e,f). Together, this suggests that there is a population of myeloid cells that, like microglia, are CD45^lo^ but do not express microglial marker P2RY12. These cells accumulate in the CNS of aged naïve mice, but may also accumulate in the CNS of younger mice after viral infection.

## 3 DISCUSSION

Several studies have reported that advanced age is associated with increased lethality in mice (Brien et al., 2009; Richner et al., 2015) and humans infected with WNV. Evidence suggests that viral titers in the brain directly correlate with mortality; however, the mechanisms underlying this increased neurovirulence in the elderly are unclear. We evaluated the impact of age on antiviral immunity within the CNS. Our results showed that aged mice had increased viral infection in peripheral tissues including kidney and serum. This increased viral replication correlated with increased percentage of PD1^+^ T cells in mock‐infected 85 week spleens and at 4 dpi, as well as reduced numbers of WNV‐specific CD8^+^ T cells. We also found that 85 week mice had reduced delays in GI transit compared with 16 week mice following infection. Previous studies have shown that GI dysmotility is due to antiviral T cells (White et al., 2018), suggesting this decreased delay in aged animals correlates with the decreased antiviral activity of T cells.

Virological control in the CNS requires coordinated cellular immunity, particularly the infiltration and activation of inflammatory monocytes and antiviral T cells (Sitati & Diamond, 2006). In the CNS, we detected higher viral replication in 85 week mice just prior to when they succumbed to infection, supporting previous data that lethality is associated with viral dissemination to the CNS.

Reduced lymphocyte response persisted after the acute viral infection had cleared. At 30 dpi, a time point at which virus is cleared, we found decreased numbers of CD8^+^ T cells within the cortices of 85 week mice compared with 16 week animals. Of the WNV‐specific CD8^+^ T cells, a greater percentage of cells isolated from 85 week cortices were positive for immune checkpoint molecule PD1, and they expressed PD1 at a higher MFI. This higher expression of PD1 on 85 week CD8^+^ T cells correlated with lower expression level of IFNγ upon *ex vivo* restimulation. Together, these data suggest there is impaired antiviral T cell response in the cerebral cortices of aged mice, and the T cells that ultimately reside in the parenchyma are less primed to respond to viral infection. Importantly, it has been reported previously that the WNV model presents variable disease outcomes and can result in survivorship bias (Suen et al., 2016; Wuertz et al., 2019). This is a critical factor to consider when interpreting acute time course versus survival end‐point experiments because animals that were euthanized at the acute time points may have progressed toward either survival or terminal disease courses. Thus, the magnitude of the differences between surviving aged versus young mice may be underrepresented compared with those aged mice that did not survive.

The detection of viral genome in the cerebral cortices by qRT‐PCR indicates neuroinvasive infection occurred in nearly all mice, including 16 week mice, which display significantly higher survival rates compared with aged animals. This is consistent with previous studies indicating that antiviral responses may clear low levels of virus from the CNS without significant injury. Younger survivors of viral encephalitis also maintain resident CD8^+^ T cells within the brain (Wakim et al., 2010), but this process is deficient in aged mice. This may be related to the limited expansion of the microglial population in 85 week cortices at 30 dpi. Considering that T cells promote microglia‐mediated synaptic elimination during recovery from flavivirus infection (Garber et al., 2019), these brain‐resident T cells could contribute to the neurocognitive difficulties that manifest in WNV‐infected patients regardless of severity of WNV infection and in the absence of clinically neuroinvasive disease (Samaan et al., 2016).

Myeloid cells including brain‐resident microglia and infiltrating macrophages are critical to CNS antiviral immunity. These cells protect the CNS from viral infection by producing antiviral cytokines, phagocytosing virus‐infected and dying neurons, and inducing neuronal repair and homeostasis (Klein et al., 2019). Myeloid cells also enhance the activation of antiviral T cells by expressing MHC molecules, costimulatory signals, and inflammatory cytokines (Durrant et al., 2013; Funk & Klein, 2019; Welten et al., 2015). We found that the cerebral cortices of 85 week mice had a greater number of CD11b^+^CD45^lo^ cells both in mock and at 9 dpi. A proportion of these cells are positive for microglia marker P2RY12, but another proportion are P2RY12‐negative, suggesting they are either atypical microglial cells or have downregulated P2RY12 in response to activation. At 30 dpi, there was a significant increase in the number of P2RY12^+^CD11b^+^CD45^lo^ microglia in 16 week but not 85 week cortex, suggesting the normal expansion of the microglial population following WNV infection does not occur in the brains of 85 week mice. Of interest, we found a significant increase in the number of P2RY12^neg^CD11b^+^CD45^lo^ cells in 16 week cortices at 30 dpi compared with mock, but no change in the 85 week cortices following infection. Of the P2RY12^neg^CD11b^+^CD45^lo^ cells, nearly all isolated from 16 week cortices at 30 dpi express CD68, verifying they are phagocytic myeloid cells. Together, these results suggest that aged cerebral cortex homeostatically accumulates this population of myeloid cells whereas younger mice only accumulate these cells after viral infection. The origin of these cells is unclear, but they may derive from either infiltrating macrophages that have downregulated CD45 expression upon resolution of the viral infection or brain‐resident microglia that have downregulated P2RY12 in response to activation (Bellver‐Landete et al., 2019; Zrzavy et al., 2017). While normal microglia are derived from yolk sac progenitor cells, monocyte‐derived progenitors can supplement the microglial pool under certain conditions such as irradiation, neurodegeneration, and brain cancers (Lund et al., 2018). It remains unclear whether these recruited cells become a part of the microglial population or are eliminated from the CNS upon resolution of the inflammatory stimulus (Sevenich, 2018). Alternatively, downregulation of homeostatic microglial signature genes, including P2RY12, has been associated with neurodegenerative diseases and during aging (Keren‐Shaul et al., 2017).

Myeloid cells are important mediators of antiviral immunity, but age‐related intrinsic changes in these cells also contribute to the systemic condition of “inflamm‐aging” (Beek et al., 2019). “Inflamm‐aging” describes the gradual increase in inflammatory signaling that occurs with aging, affecting nearly all cells and promoting overall age‐related decline in normal function. With age, microglia numbers and density increase, along with deterioration of their regular order and distribution (Tremblay et al., 2012). “Dystrophic microglia” refers to the morphologic changes in aged microglial, including shorter dendritic processes that are more variable in size and lack circular symmetry (Tremblay et al., 2012). Functionally, aged microglia exist in a state of increased basal activation and are primed to react in an amplified and prolonged manner when challenged (Zhao et al., 2019). Consistent with this, we found increased expression of the phagocytic marker CD68 in microglia of both mock‐ and post‐infectious 85 week brain, which is consistent with reports of age‐associated increases in microglial CD68^+^ lysosome enlargement (O'Neil et al., 2018). Together, our data suggest that peripheral infection with WNV promotes the accumulation of myeloid cells that harbor an aged phenotype in the cortices of 16 week mice. Further studies examining aging programs and the long‐term impact of activated microglia are warranted.

We found increased TUNEL‐positive perinuclear punctae in neurons of 85 week versus 16 week mice at 9 dpi. Lan et al. showed that aged cells traffic damaged DNA to the cytosol, which then activates inflammatory pathways such that aged cells upregulated genes associated with IFN‐α response, TNF‐α signaling, and IL‐6 signaling (Lan et al., 2019). This may contribute to the increased basal inflammation seen here in aged versus 16 week microglia.

In summary, our study provides evidence that aged mice are more susceptible to lethal WNV infection due to decreased antiviral responses to WNV infection in the CNS leading to uncontrolled viral replication. We also observed low levels of viral genome in nearly all 16 week mouse cortices, which was associated with robust antiviral T cell infiltration and microglia expansion at a time point after the acute infection had resolved. Given the role of T cells in driving microglia‐mediated synaptic elimination, this response may be important in understanding post‐infectious cognitive sequelae in WNV‐infected patients that lack clinical neuroinvasive disease.

## 4 EXPERIMENTAL PROCEDURES

### 4.1 Ethics statement

All experiments were performed in compliance with the recommendations in the Guide for the Care and Use of Laboratory Animals of the National Institutes of Health and according to the international Guiding Principles for Biomedical Research Involving Animals. The protocol was approved by the Washington University School of Medicine in St Louis Animal Safety Committee (#20170064).

### 4.2 Viruses

WNV‐NY is strain 3000.0259, isolated in New York in 2000 and passaged twice in C6/36 *Aedes albopictus* cells to generate an insect cell‐derived stock for subcutaneous inoculations in the footpad. Stock titers for all viruses were determined using BHK21 cells for viral plaque assay as previously described (Brien et al., 2013).

### 4.3 Mouse experiments

All mice used in these experiments were male C57BL/6J inbred mice obtained commercially (The Jackson Laboratory). All mice were housed in pathogen‐free facilities at the Washington University School of Medicine. For viral inoculations, mice were anesthetized with a cocktail of ketamine/xylazine/acepromazine and then inoculated subcutaneously in the footpad with 100 PFU in 50 μl inoculum. Mock‐infected animals were anesthetized and then injected in the footpad subcutaneously with 50 μl virus diluent (1% FBS in HBSS).

### 4.4 Viral burden measurements

Mice were infected with WNV and euthanized at specific days post‐infection, as indicated. For tissue collection, mice were deeply anesthetized with ketamine/xylazine/acepromazine, blood collected via heart stick in serum separator tubes and then perfused with 20 ml of sterile Dulbecco's phosphate‐buffered saline (dPBS; Gibco). The spleen and kidneys were collected, and then the brain and spinal cord were removed and microdissected. All organs were snap frozen on dry ice, weighed, and then homogenized in 500 μl sterile dPBS. Virus was titered by standard plaque assay with BHK21 cells (Brien et al., 2013). Viral RNA in the serum and cortex was isolated by Qiagen QIAamp Viral RNA Mini Kit and RNeasy Mini Kits, respectively. Viral genome was quantified using a standard curve of known viral titer and the following TaqMan quantitative RT‐PCR (qRT‐PCR) primers and probe, as described previously (Brien et al., 2013): Forward: 5′‐TCA GCG ATC TCT CCA CCA AAG‐3′; Reverse: 5′‐GGG TCA GCA CGT TTG TCA TTG‐3′; Probe: 5′‐/56‐FAM/TGC CCG ACC ATG GGA GAA GCT C/36‐TAMSp/‐3.

### 4.5 GI motility measurements

In vivo bowel motility was assessed by passage of carmine red dye as described (Avetisyan et al., 2015; White et al., 2018). Whole bowel transit time was determined by administration of 300 μl of 6%(w/v) carmine red dye (Sigma‐Aldrich) dissolved in distilled water containing 0.5% methyl cellulose (Sigma‐Aldrich) by oral gavage. Gavaged mice were placed in individual containers with white bottoms and checked at 10‐min intervals through 6 h for the passage of carmine‐containing, red fecal pellets.

### 4.6 Leukocyte isolation and flow cytometry

For flow cytometry experiments, leukocytes were isolated as described previously (Funk & Klein, 2019). Mice were deeply anesthetized with ketamine/xylazine/acepromazine and then transcardially perfused with 20 ml dPBS, and the spleen, lymph nodes, and brain were removed. Brain tissue was minced and digested in a HBSS (Gibco) containing 0.05% collagenase D (Sigma), 0.1 μg/ml TLCK trypsin inhibitor (Sigma), 10 μg/ml DNase I (Sigma), and 10 mM Hepes pH 7.4 (Gibco) for 1 h at 22°C with shaking. Brain, spleen, and LN tissues were pushed through a 70‐μm strainer and centrifuged at 500 *g* for 10 min. Brain cell pellets were resuspended in 37% isotonic Percoll (GE healthcare) and centrifuged at 1200 *g* for 30 min to remove myelin debris, and the pellet was resuspended in dPBS. In spleen samples, red blood cells were lysed with ACK lysing buffer (Gibco) for 5 min, then centrifuged at 500 g for 10 min, and resuspended in dPBS. For *ex vivo* restimulation, isolated cells were then treated with 1 μg/ml ionomycin and 5 ng/ml phorbol myristate acetate to stimulate cytokine expression and 5 μg/ml Brefeldin A to block cytokine exocytosis for 4 h at 37°C, 5% CO_2_. Prior to immunostaining, all cells were blocked with 1:50 TruStain FcX anti‐mouse CD16/32 (Clone 93, Biolegend, Cat 101320) for 5 min. Cells were stained with Live/Dead Fixable Blue at 1:1000 following dissolution in 40 μl DMSO (Invitrogen, L34962) and extracellular antibodies, as indicated, for 15 min at 22°C, then washed twice with dPBS, fixed, and permeabilized with Foxp3/Transcription Factor Staining Kit (eBioscience, 00–5523–00). Intracellular markers were stained with antibodies, as indicated for 15 mins, and then cells were washed twice with permeabilization buffer and twice with dPBS and then fixed with 2% paraformaldehyde (PFA). Data were collected with a BD LSRFortessa X‐20 flow cytometer and analyzed with FlowJo software.

### 4.7 Flow cytometry antibodies and tetramers

All antibodies used at 1:200: CD11b (Clone M1/70, Biolegend, Cat 10137), CD45 (Clone 30‐F11, eBioscience, Cat 56–0451–82), MHCII (Clone M5/114.15.2, Biolegend, Cat 107626), CD80 (Clone 16‐10A1, Biolegend, Cat 104706), CD103 (Clone 2E7, eBioscience, Cat 48‐1031‐80), CD8a (Clone 53‐6.7, Biolegend, Cat 100712), CD4 (Clone RM4‐5, BD Biosciences, Cat 550954), CD44 (Clone IM7, Biolegend Cat 103011), P2RY12 (Clone S16007D, Biolegend Cat 848006), IFNγ (Clone XMG1.2, Biolegend Cat 505826), CD68 (Clone FA‐11, Biolegend Cat 137017). WNV‐specific CD8+ T cells were identified with fluorescent‐labeled immunodominant Db‐restricted NS4B peptide.

### 4.8 TUNEL staining, immunohistochemistry, and confocal microscopy

At specific time points, mice were perfused with 20 ml cold PBS, then brains were removed and immersion‐fixed overnight in 4% PFA. After cryoprotection in two exchanges of 30% sucrose, brains were frozen in OCT, and ten micrometer sagittal sections were cut. For tissue stained with TUNEL reagent, sections were washed with PBS and permeabilized with 0.1% Triton X‐100/0.1% sodium citrate. TUNEL staining reagent was prepared according to manufacturer's directions (Roche in situ cell death kit, TMR Red, Cat 12‐156‐792‐910). Sections then were immunostained with antibodies against NeuN (1:400, Millipore, Cat ABN90P) and Iba1 (1:500, Synaptic, Cat 234006) by blocking sections with 5% goat serum/0.1% Tween‐20/PBS and then incubated overnight at 4°C with anti‐NeuN and anti‐Iba1. Sections were washed with 0.1% Tween‐20/PBS and incubated with fluorescently labeled secondary antibodies, counterstained with 1 μg/ml DAPI, and coverslipped with Prolong Gold Antifade Mountant. For tissue immunostained with Iba1 and CD68 (1:200, BioRad, Cat MCA341R) or NeuN, sections were permeabilized with 0.1% Triton X‐100 and then blocked with 5% goat serum/0.1% Triton X‐100. Sections were incubated overnight at 4°C with anti‐Iba1 and anti‐CD68. Sections were washed with 0.1% Tween‐20/PBS and incubated with fluorescently labeled secondary antibodies, counterstained with 1 μg/ml DAPI, and coverslipped with Prolong Gold Antifade Mountant. All images were captured using a Zeiss LSM 510 laser scanning confocal microscope at 20X objective magnification. The number of TUNEL‐positive punctae was counted using ImageJ. Area covered by each Iba1 and CD68 and the Manders coefficient for colocalization were quantified using ImageJ.

### 4.9 Statistical analysis

Statistical analyses were performed using Prism 8.0 (GraphPad Software). All data were analyzed using an unpaired t test or two‐way ANOVA with Sidak's post‐test to correct for multiple comparisons, as indicated in the corresponding figure legends. A *p* value <0.05 was considered significant.

## CONFLICT OF INTEREST

M.S.D. is a consultant for Inbios, Vir Biotechnology, NGM Biopharmaceuticals, and on the Scientific Advisory Board of Moderna. The Diamond laboratory has received unrelated funding support in sponsored research agreements from Moderna, Vir Biotechnology, and Emergent BioSolutions.

## AUTHOR CONTRIBUTIONS

KEF, AA, PD, JPW, MSD, and RSK contributed to experimental design and data analyses. KEF, AA, PD, JPW, ALS, and SFR performed experiments and generated figures. KEF, AA, MSD, and RSK contributed to the writing and editing of the manuscript.

## Supporting information

Fig S1

Fig S2

Fig S3

Fig S4

Fig S5

Fig S6

Fig S7

Fig S8

## Data Availability

The data that support the findings of this study are available from the corresponding author upon reasonable request.
