# Supplementary figures and images for: Decreased antiviral immune response within the central nervous system of aged mice is associated with increased lethality of West Nile virus encephalitis
Source: Aging Cell. 2021 Jul 30;20(8):e13412. doi: 10.1111/acel.13412 (PMC8373274; doi:10.1111/acel.13412)

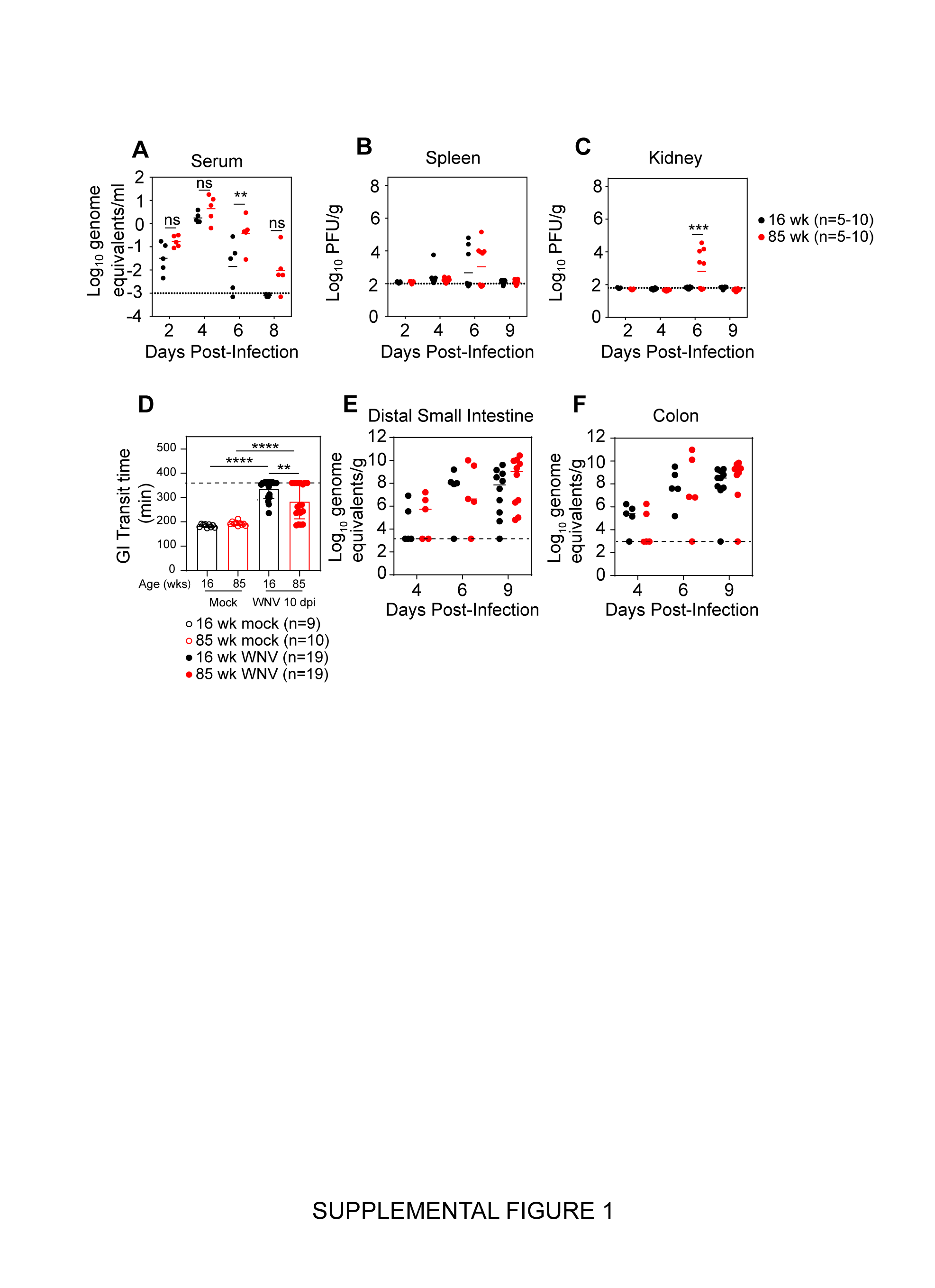

Supplement: Supplementary file 1 — Fig S1 [file ACEL-20-e13412-s007.tif]

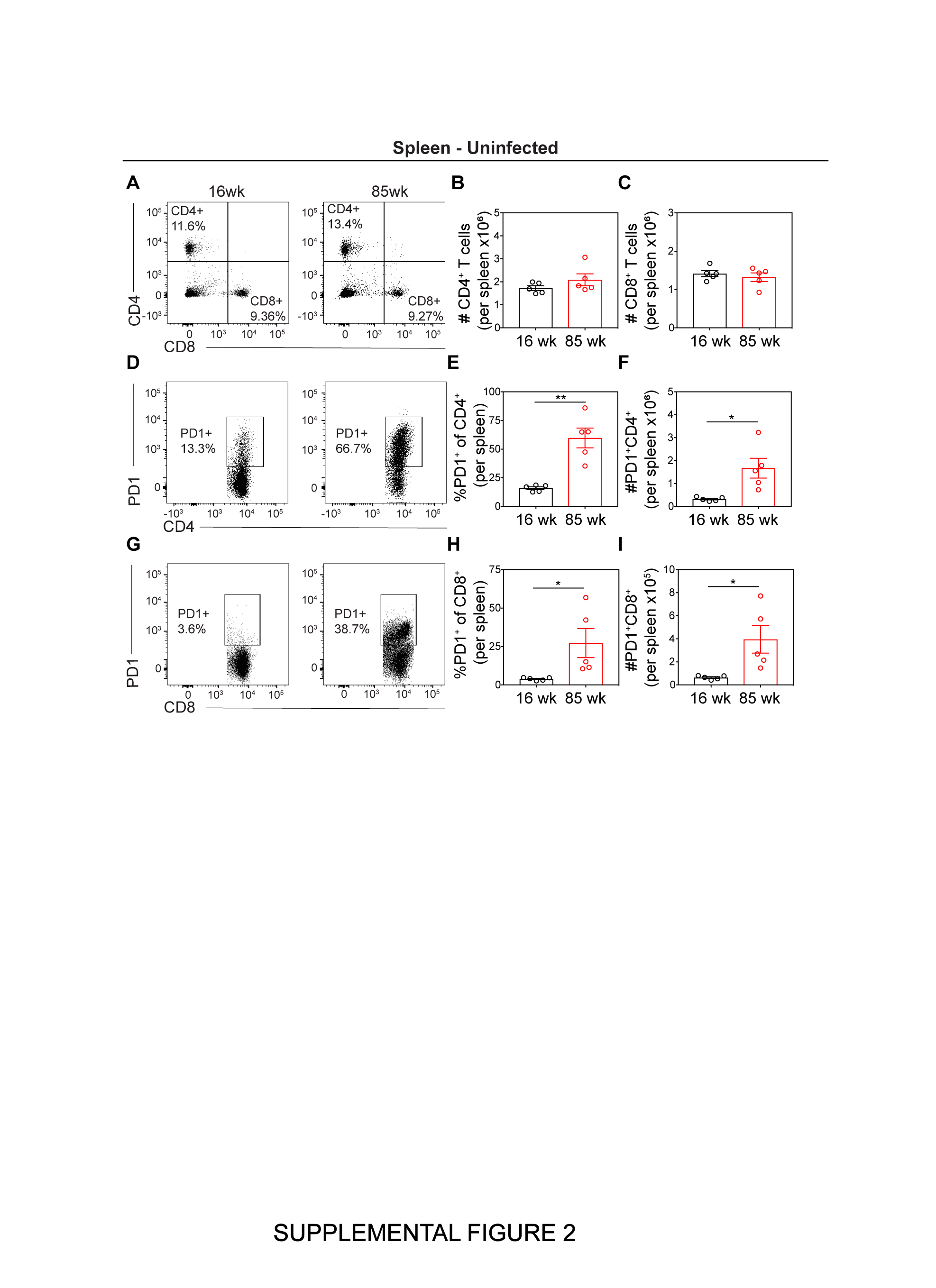

Supplement: Supplementary file 2 — Fig S2 [file ACEL-20-e13412-s004.tif]

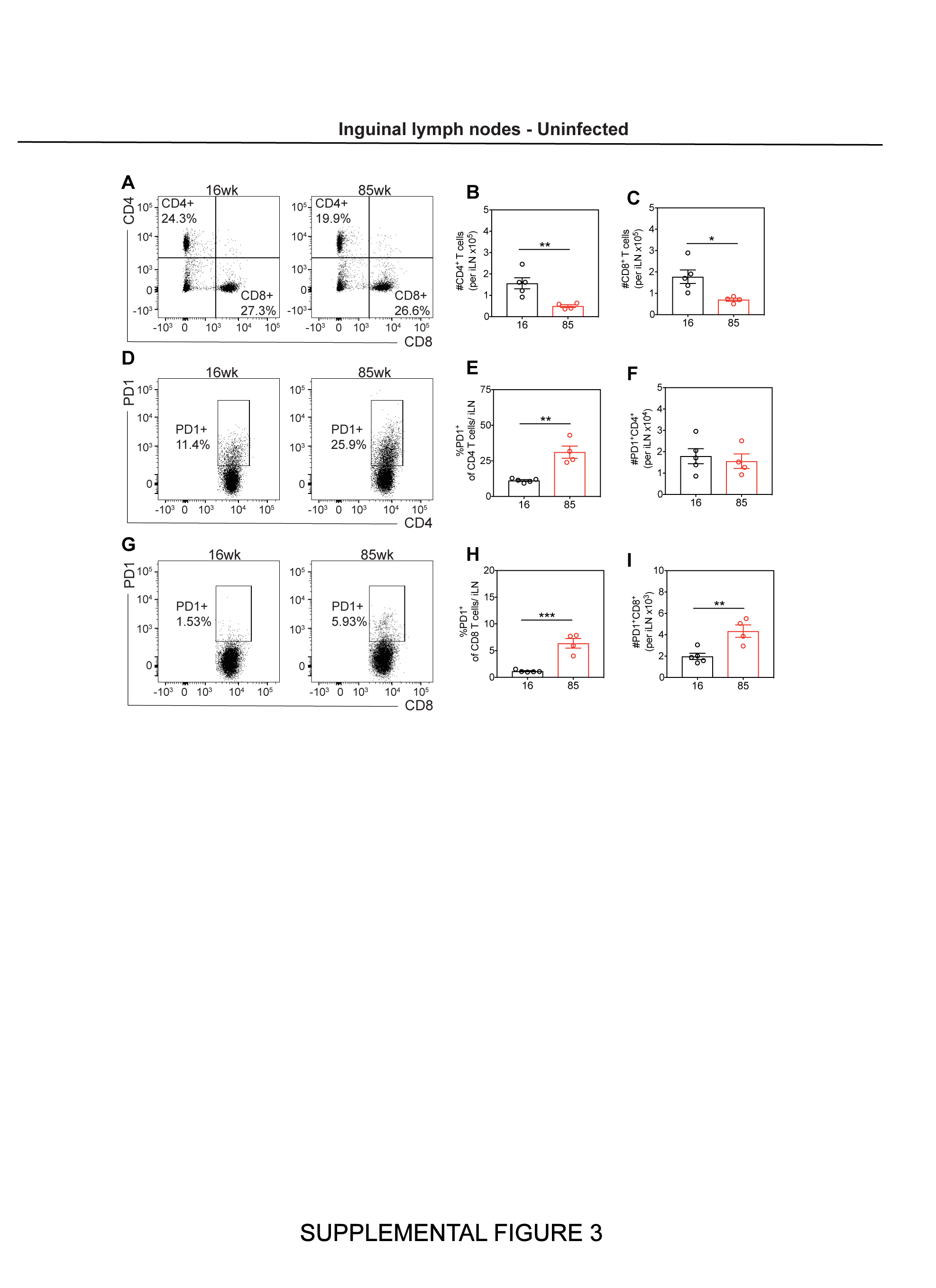

Supplement: Supplementary file 3 — Fig S3 [file ACEL-20-e13412-s001.tif]

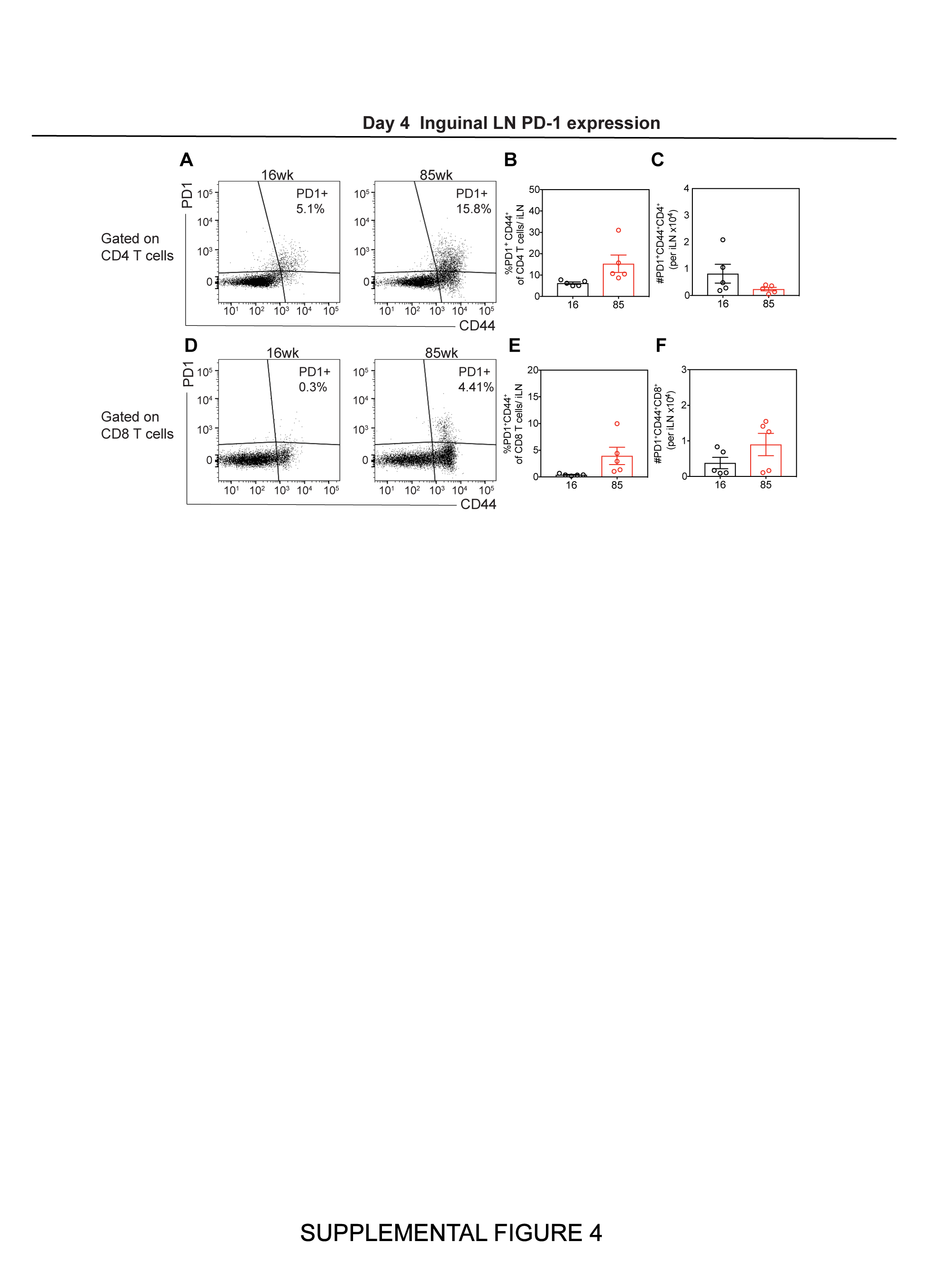

Supplement: Supplementary file 4 — Fig S4 [file ACEL-20-e13412-s002.tif]

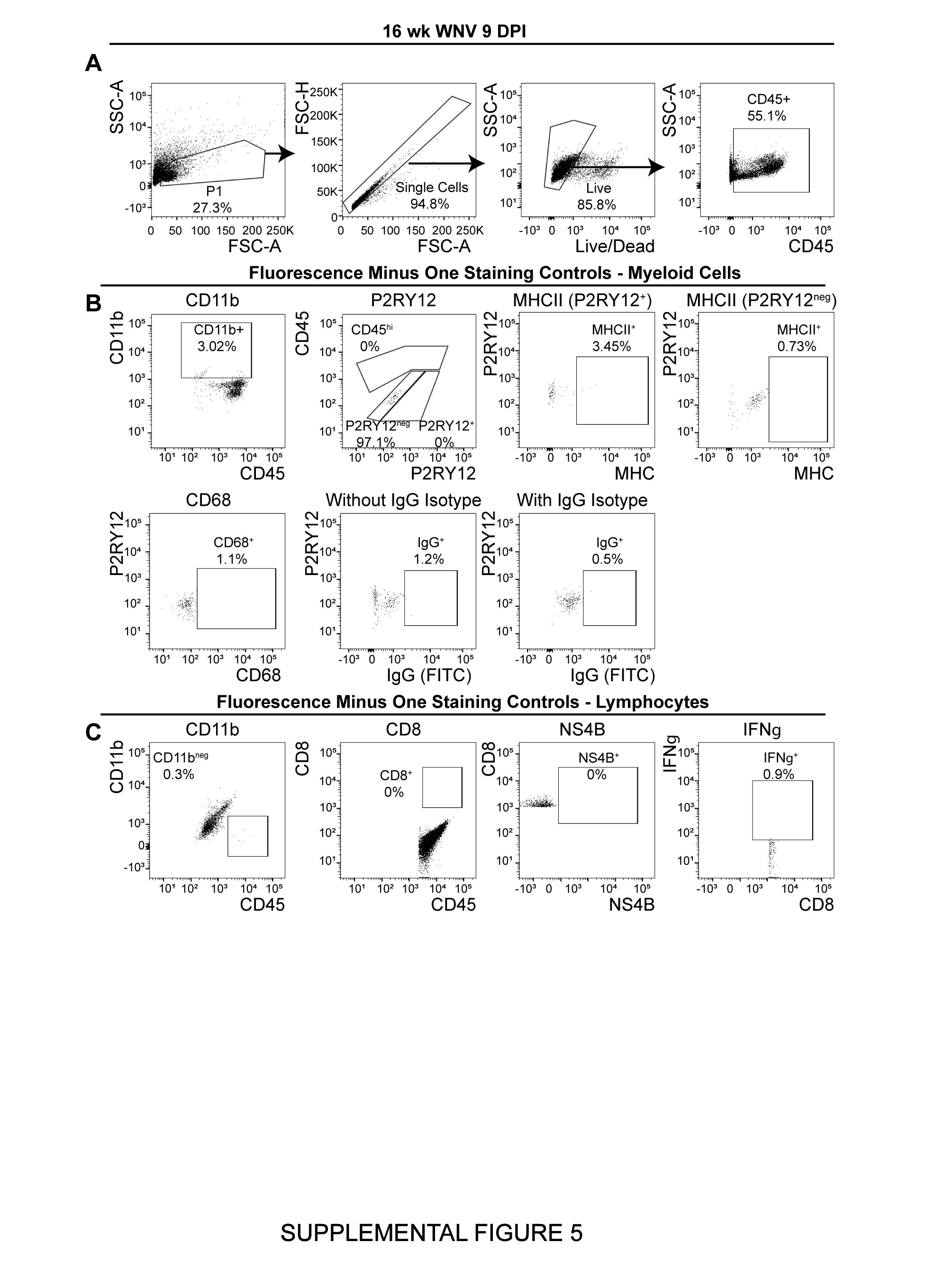

Supplement: Supplementary file 5 — Fig S5 [file ACEL-20-e13412-s003.tif]

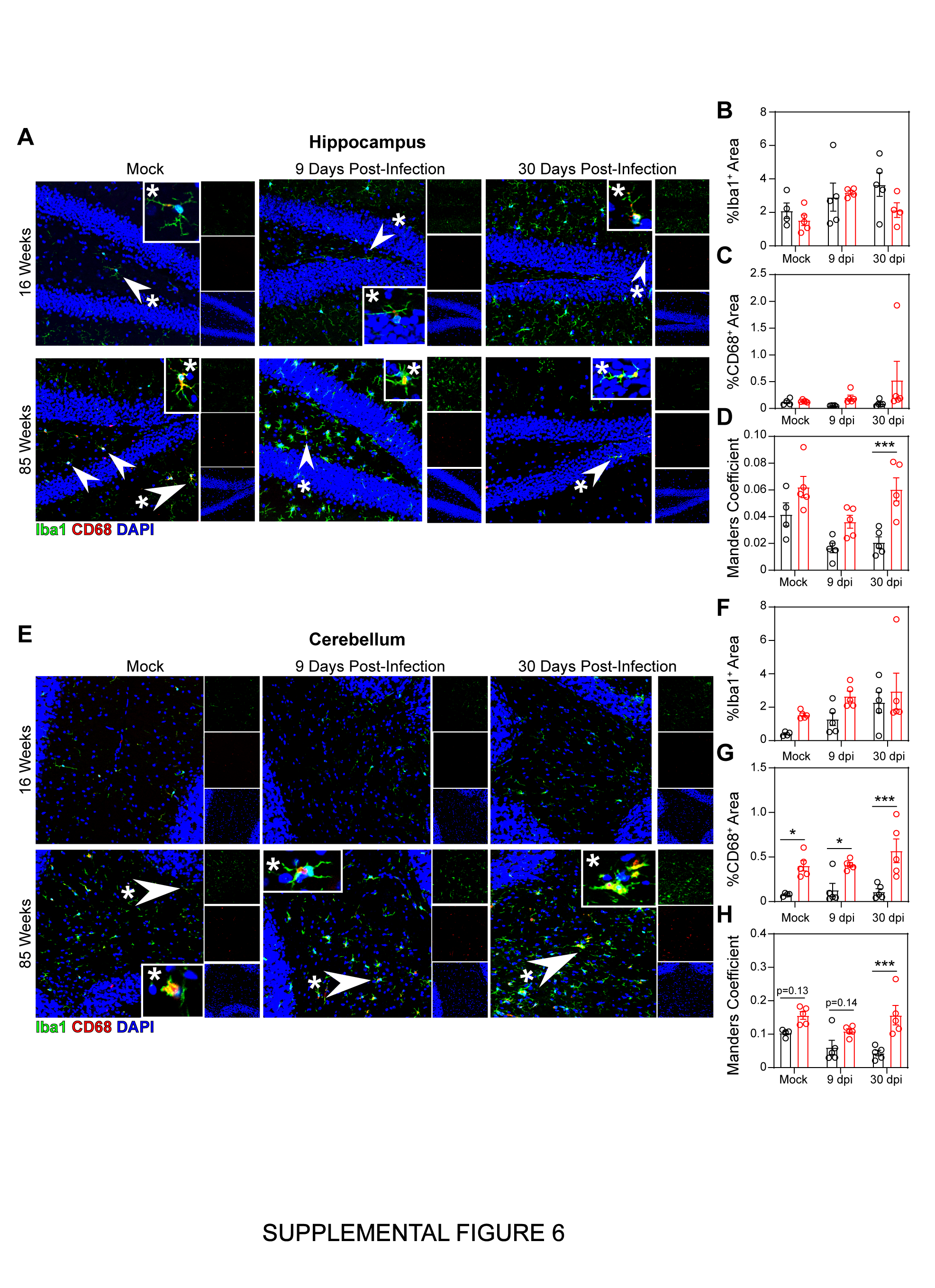

Supplement: Supplementary file 6 — Fig S6 [file ACEL-20-e13412-s006.tif]

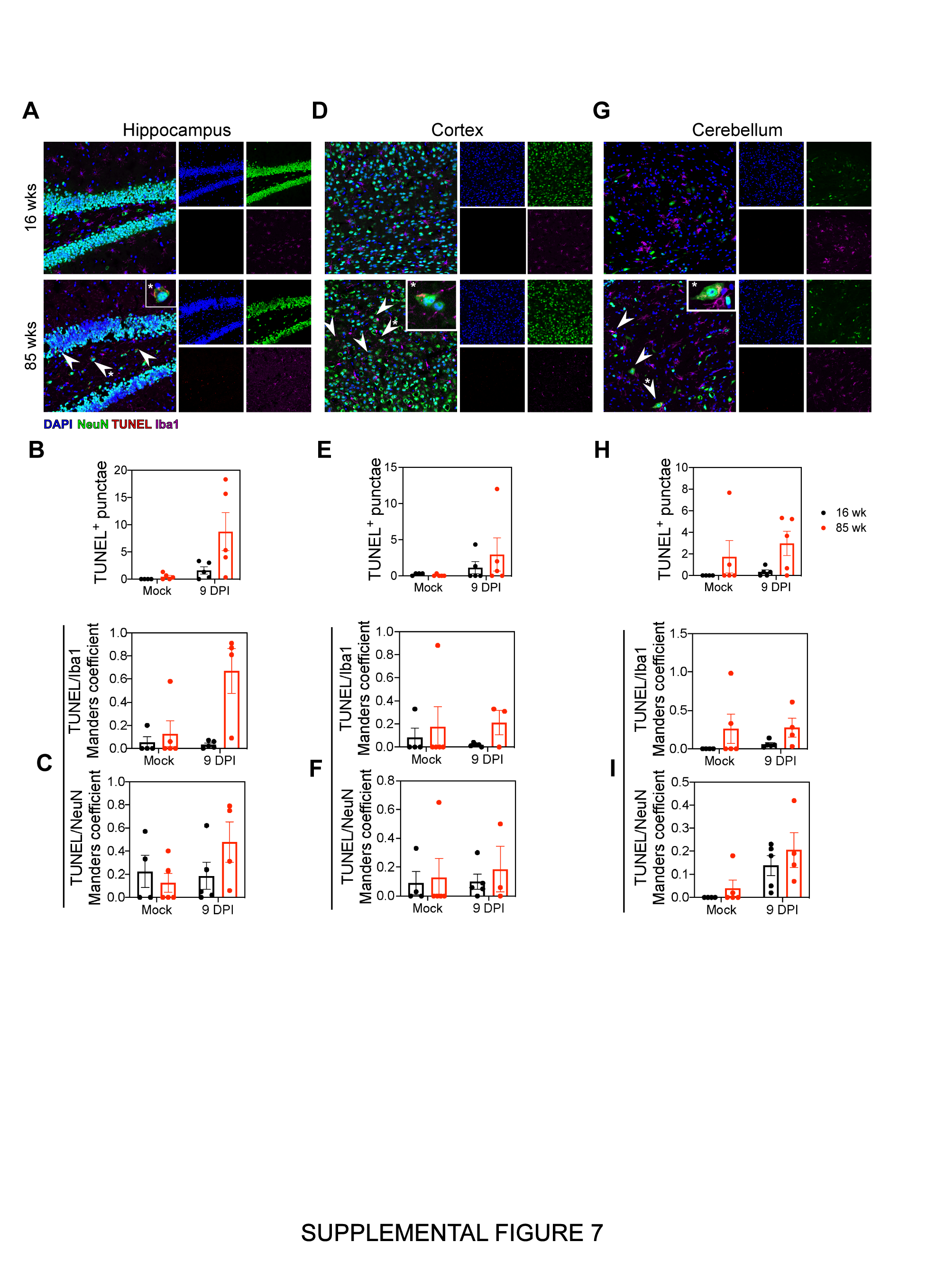

Supplement: Supplementary file 7 — Fig S7 [file ACEL-20-e13412-s008.tif]

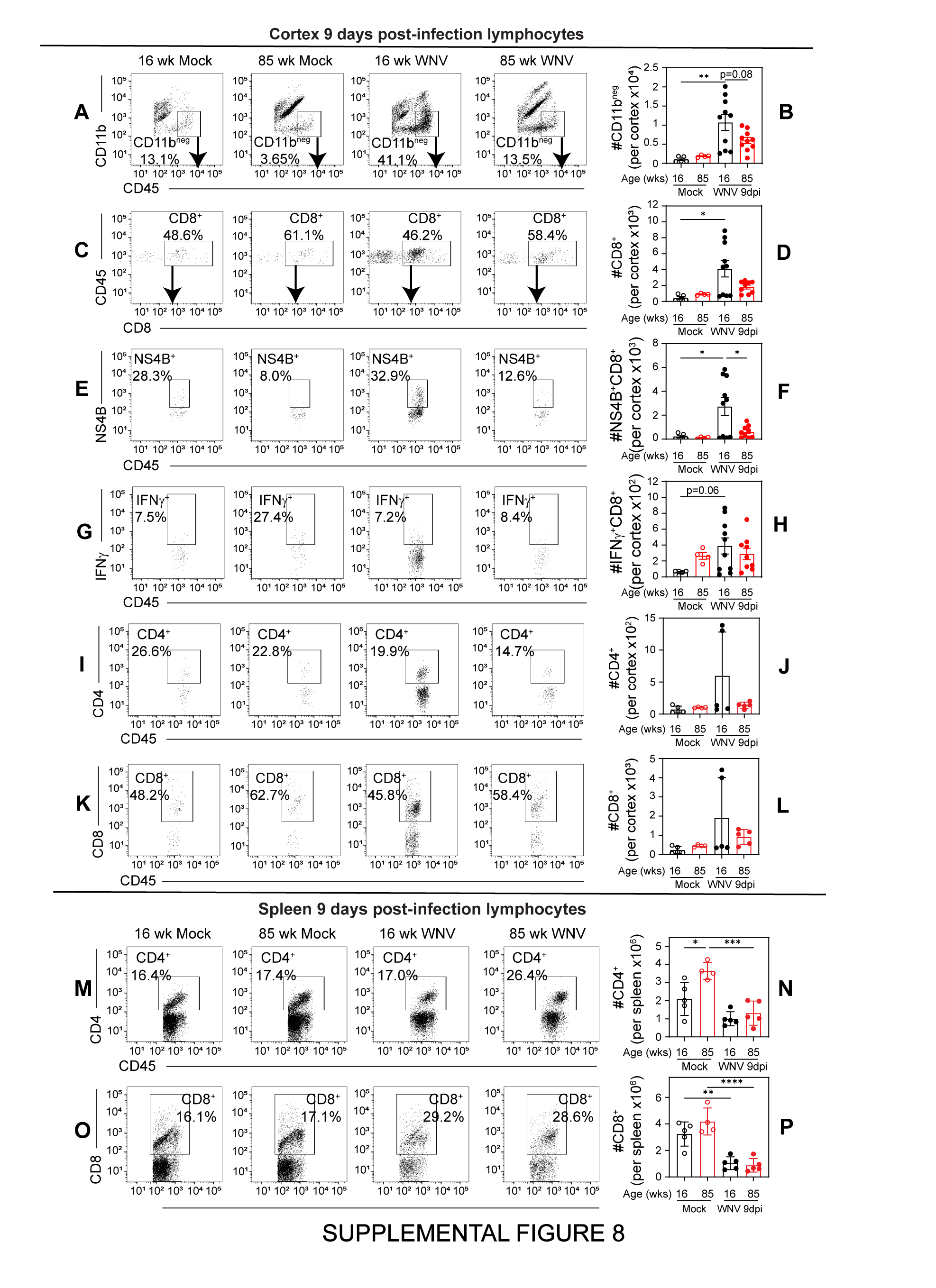

Supplement: Supplementary file 8 — Fig S8 [file ACEL-20-e13412-s005.tif]
